# Physical Activity and Depression in Breast Cancer Patients: Mechanisms and Therapeutic Potential

**DOI:** 10.3390/curroncol32020077

**Published:** 2025-01-29

**Authors:** Anlong Li, Xinyi Zheng, Dajie Liu, Runze Huang, Han Ge, Ling Cheng, Mingjun Zhang, Huaidong Cheng

**Affiliations:** 1 Department of Oncology, The Second Affiliated Hospital of Anhui Medical University, Hefei 230601, China; lianlong1966@163.com (A.L.); 15574923341@163.com (D.L.); 18756909618@163.com (R.H.); gh144621@126.com (H.G.); 2 The Second School of Clinical Medicine, Anhui Medical University, Hefei 230032, China; 3 The Third School of Clinical Medicine, Southern Medical University, Guangzhou 510500, China; xyzheng_jul@163.com; 4 Department of Oncology, Shenzhen Hospital of Southern Medical University, Shenzhen 518000, China; 5 School of Nursing, Anhui Medical University, Hefei 230032, China; 6 Department of Oncology, Shenzhen Hospital of Guangzhou University of Chinese Medicine (Futian), Shenzhen 518000, China; chenglingnjzyydx@sina.com

**Keywords:** breast cancer, depression, physical activity, inflammation, neuroplasticity

## Abstract

Breast cancer is a significant traumatic experience that often leads to chronic stress and mental health challenges. Research has consistently shown that physical activity—especially exercise—can alleviate depressive symptoms; however, the specific biological mechanisms underlying these antidepressant effects remain unclear. In this review, we comprehensively summarize the biological mechanisms of depression and the antidepressant mechanisms of physical activity and explore the biological processes through which exercise exerts its antidepressant effects in breast cancer patients. We focus on the impact of physical activity on inflammation, the endocrine system, glutamate, and other aspects, all of which play crucial roles in the pathophysiology of depression. Moreover, we discuss the heterogeneity of depression in breast cancer patients and the complex interactions between its underlying mechanisms. Additionally, we propose that a deeper understanding of these mechanisms in the breast cancer population can guide the design and implementation of exercise-based interventions that maximize the antidepressant benefits of physical activity. Finally, we summarize the current research and propose future research directions.

## 1. Introduction

Breast cancer is one of the most common malignancies among women, with patients not only enduring physical pain but also facing significant psychological stress. This stress often arises from fears related to survival, changes in body image, and the side effects of treatment, including postoperative pain, fatigue, and discomfort associated with chemotherapy and radiotherapy [1]. These psychological burdens severely impact patients’ quality of life and may interfere with their treatment adherence, potentially lowering their survival rate [2,3]. The incidence of depression significantly increases during the diagnosis and treatment of breast cancer. Studies have shown that the prevalence of depression among breast cancer patients is as high as 32.2%, much higher than in the general population [4]. Depression is a global mental health issue that affects millions of people, particularly women. According to the World Health Organization, approximately 280 million people worldwide suffer from depression, with a notably higher prevalence in women—about 50% higher than in men [5]. Symptoms of depression include persistent low mood, insomnia, changes in appetite, difficulty concentrating, and, in severe cases, even suicidal ideation. Notably, in breast cancer patients, depression is not merely a psychological response but also involves complex biological mechanisms. For instance, endocrine therapies used in breast cancer treatment can cause significant fluctuations in estrogen levels. Estrogen may contribute to oxidative stress in breast cancer cells by inhibiting uncoupling proteins, thus influencing the onset and progression of depression [6]. Additionally, chronic inflammation induced by cancer and its treatments may exacerbate depressive symptoms through pro-inflammatory cytokines such as IL-6 and TNF-α [7,8]. Therefore, understanding the biological basis of depression and its treatment in breast cancer patients is of critical clinical importance for improving their overall prognoses.

Common treatments for depression include pharmacological therapies, cognitive behavioral therapy (CBT), psychological interventions, and physical activity [9]. Depression is a highly heterogeneous disorder, with different individuals exhibiting varying symptoms and pathological mechanisms. This diversity challenges the universal effectiveness of traditional pharmacological and psychological treatments, making it difficult to achieve effective treatment for all patients. Research indicates that approximately 30–40% of patients with depression show resistance to antidepressants, a condition known as treatment-resistant depression, complicating clinical management [10]. Moreover, while CBT has demonstrated effectiveness in some patients, its efficacy is often influenced by individual psychological traits, symptom types, and the duration of the illness [11]. These individual differences not only affect treatment outcomes directly but also highlight the need for more personalized treatment approaches. The limitations of traditional therapies have spurred the exploration of new treatment strategies, including precision medicine and personalized treatment pathways [12]. Through deeper investigation into the complexity and pathological background of depression, scientists may develop more targeted and comprehensive treatments, enhancing both their efficacy and reliability. These findings underscore the continued importance of pharmacological and psychological treatments in mental health care, while also emphasizing the growing need for innovative treatment strategies to address the heterogeneity and challenges of depression more effectively.

Among various interventions for depression in breast cancer patients, physical activity is particularly notable due to its significant protective and therapeutic effects on depression. A meta-analysis of 49 prospective cohort studies demonstrated that physical activity significantly reduces the risk of depression, with benefits observed across different age groups, including adolescents, adults, and the elderly. Those with higher levels of physical activity had a 17–22% lower risk of developing depression [13]. Mendelian randomization results indicate a negative correlation between objectively measured physical activity and the risk of depression, suggesting that increasing physical activity levels can significantly reduce the risk of depression [14]. A longitudinal biobank study involving nearly 8000 adults found that even in individuals with a high genetic risk for depression, increased physical activity effectively reduced the incidence of depression. This suggests that physical activity offers a protective effect against depression, even in genetically predisposed populations [15]. Evidence has also been established in breast cancer patients. A meta-analysis of randomized controlled trials on breast cancer survivors revealed that moderate-intensity physical activity, particularly aerobic exercise, significantly alleviated depressive symptoms. The interventions typically lasted 12 weeks, with at least 135 min of exercise per week leading to notable improvements in depressive symptoms [16]. Physical activity indirectly improved depressive symptoms by reducing fatigue and enhancing daily functioning, independent of other psychological interventions [17]. In addition to alleviating depressive symptoms, physical activity provides numerous other benefits for breast cancer patients. Observational studies have shown a positive correlation between increased physical activity and survival rates among breast cancer survivors, with moderate physical activity reducing the risk of breast-cancer-related mortality and potentially extending overall survival [18].

Increasing physical activity helps alleviate common side effects of breast cancer treatment, such as joint pain, fatigue, and muscle weakness, thus improving patients’ ability to tolerate treatment and enhancing recovery outcomes [19]. Physical activity also plays a crucial role in cardiovascular health, lowering the risk of cardiovascular disease. This is especially important for early-stage breast cancer patients undergoing cardiotoxic treatments [20]. While clinical studies often employ exercises, yoga, Tai Chi, and other methods to increase physical activity, the exact mechanisms by which physical activity exerts antidepressant effects—particularly in breast cancer patients—remain unclear. Compared to systematic reviews, non-systematic reviews offer greater flexibility. During the literature selection process, classic studies can be appropriately included, and innovative perspectives and methods can be integrated based on the specific needs of the research. Moreover, the impact of physical activity on depression in breast cancer patients is an interdisciplinary research area, involving biology, psychology, exercise science, and other disciplines, encompassing various types of studies (such as clinical research, laboratory studies, and retrospective analyses). A non-systematic review allows for the flexible selection of different types of studies in the literature, which helps present the diversity of the field and enriches the research background and perspectives.

In this review, we selected the literature related to depression and physical activity in breast cancer patients, using keywords such as “breast cancer”, “depression”, “physical activity”, “exercise”, “mental health”, “cancer-related fatigue”, “psychological well-being”, “physical activity interventions”, and “impact of exercise on depression” for the literature search. The time frame for the selected studies primarily focused on the past 10 years to ensure the timeliness of the information. During the selection process, we prioritized studies that provide mechanistic explanations or have high academic impact, particularly those exploring the antidepressant mechanisms, intervention effects, and psychophysiological mechanisms of physical activity in breast cancer patients. Through manual searching and citation tracking, we further included some classic studies and key papers to ensure the breadth and depth of this review. The final selected literature is closely related to the topic of this review and provides solid theoretical support. This review will comprehensively summarize the key biological mechanisms underlying the antidepressant effects of physical activity. We conducted a non-systematic literature search to identify potential mechanisms through which physical activity influences depression in breast cancer patients. Notably, due to a lack of research specifically focused on breast cancer patients in certain areas, findings from non-cancer populations and animal studies will be included when necessary. In clinical practice, exercise is the most common method used to increase physical activity, and therefore, the discussion will primarily focus on exercise.

## 2. Biological Mechanisms of Depression and the Crucial Role of Physical Activity

Breast cancer, the most common cancer among women worldwide, often subjects patients to both physical and psychological stress during treatment. Psychological issues, such as depression and anxiety, are highly prevalent among breast cancer patients and can significantly impact treatment outcomes and overall quality of life. Physical activity, in its broadest sense, refers to any form of bodily movement that results in energy expenditure through muscle activity. This includes not only structured exercise but also everyday activities such as walking and household chores. As our understanding of its health benefits has deepened, physical activity has been shown to have significant positive effects on both physical and mental health, particularly for cancer patients. For breast cancer patients, research indicates that regular physical activity not only improves physiological function and alleviates treatment-related side effects but also significantly reduces depressive symptoms and enhances overall mental health. However, the impact of physical activity on depression is not solely mediated through psychological mechanisms; rather, it also involves various biological pathways that play a crucial role. This section will explore how physical activity improves depressive symptoms in breast cancer patients through the modulation of biological pathways, including inflammation, glutamate metabolism, the neuroendocrine system, neuroplasticity, oxidative stress, and monoamine neurotransmitters. These biological mechanisms are central to the onset and progression of depression, and understanding them provides a more comprehensive perspective on the antidepressant effects of physical activity, offering scientific evidence to support the rehabilitation of breast cancer patients.

### 2.1. Inflammation

Chronic inflammation is closely linked to depression. In breast cancer patients, elevated levels of inflammatory markers and pro-inflammatory cytokines have been shown to significantly correlate with depressive symptoms. The higher the level of inflammation, the more likely it is that negative emotional biases will develop [21]. Interferon (IFN)-α, a cytokine produced by the immune system, plays a critical role in antiviral responses and immune regulation [22]. IFN-α can stimulate the production of pro-inflammatory cytokines, and in certain studies, it has been used to simulate a chronic inflammatory environment. Although IFN-α is commonly used to treat diseases such as hepatitis C and certain cancers, it has also been shown to induce depression in a significant proportion of patients. At high doses, 50% of patients without depressive symptoms will meet the criteria for major depressive disorder within three months of treatment, and a larger proportion will exhibit one or two noticeable depressive symptoms [23,24]. Studies have found that IFN-α-induced increases in IL-6 and tumor necrosis factor (TNF)-α signaling are associated with worsening depressive symptoms [25,26]. Anti-inflammatory treatments have demonstrated significant efficacy in alleviating depressive symptoms in some patients, further suggesting the bidirectional relationship between inflammation and depression, in which depression can exacerbate inflammation [27]. Depressed patients typically have elevated levels of inflammatory markers, and prolonged depression can lead to persistent immune system activation, further impairing neuronal function and establishing a vicious cycle [28].

The sources of inflammation in depressed patients are multifactorial, with psychosocial stress being a major contributor. Stress can activate both the sympathetic and parasympathetic nervous systems, promoting inflammation [29]. Poor lifestyle choices, altered gut permeability, chronic diseases, genetic factors, and environmental influences all contribute to the onset and maintenance of chronic inflammation [30]. Interestingly, while inflammatory mediators in depressed individuals are generally higher than in non-depressed individuals, the increase in pro-inflammatory cytokines and other inflammatory factors observed in depression is much less pronounced compared to autoimmune or infectious diseases. Research on depression has shown that individuals with certain characteristics, such as body mass index (BMI) and gender, may exhibit higher levels of inflammation, and after controlling for these factors, some studies indicate that there are no significant differences in inflammatory markers between depressed and non-depressed groups. Raison et al. suggested in their review that the elevated levels of multiple inflammatory biomarkers in depressed individuals are primarily due to a higher proportion of individuals within the depressed group showing these elevations compared to control populations [31].

**Figure 1:**
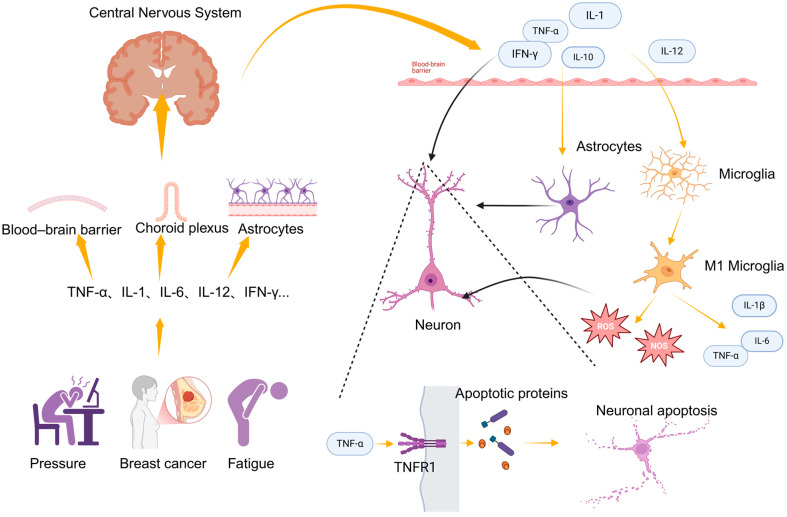
Primary pathways linking inflammation to depression. The figure was created in BioRender at the following site: https://BioRender.com.

Inflammatory factors can enter or indirectly affect the central nervous system (CNS) through several mechanisms, leading to neuroinflammation. Chronic peripheral inflammation can damage the blood–brain barrier (BBB), increasing its permeability and allowing inflammatory cells to enter the CNS [32], thereby activating neuroinflammation. Peripheral inflammatory cytokines can also enter cerebrospinal fluid through vulnerable regions, such as the choroid plexus [33], or they can act on endothelial cells and astrocytes near the BBB, indirectly activating neuroinflammation [34]. Notably, inflammatory cytokines that enter the CNS activate glial cells, with astrocytes and microglia being particularly important. Astrocytes support neurons metabolically by regulating neurotransmitter uptake and metabolism [35] but the excessive activation of astrocytes can lead to dysfunction and toxicity in neurons [36]. Microglia, the primary immune cells in the CNS, become activated when stimulated by inflammatory cytokines, altering their morphology and releasing additional pro-inflammatory cytokines (e.g., IL-1β, TNF-α, and IL-6), further exacerbating local inflammation [37]. During activation, microglia and astrocytes generate large amounts of reactive oxygen species (ROS) and reactive nitrogen species (RNS), which can directly damage cellular organelles, proteins, and DNA within neurons, leading to cellular damage, dysfunction, and even neuronal apoptosis [36]. Inflammatory cytokines can also directly interact with neurons; for example, cytokines such as TNF-α can activate apoptotic signaling pathways through receptors like TNFR1, initiating neuronal apoptosis and contributing to depressive-like behavior [38,39]. Inflammatory cytokines have been shown to increase the expression and function of serotonin (5-HT), noradrenaline, and dopamine reuptake pumps (transporters) through signaling pathways, including the mitogen-activated protein kinase (MAPK) pathway, such as p38 MAPK. This process is illustrated in Figure 1.

Inflammatory cytokines also influence serotonin synthesis by activating indoleamine 2,3-dioxygenase (IDO) [40]. These cytokines disrupt tetrahydrobiopterin, an essential cofactor for tryptophan hydroxylase and tyrosine hydroxylase, which are rate-limiting enzymes in serotonin and dopamine (as well as noradrenaline) synthesis, thereby affecting dopamine and serotonin metabolism [41]. Previous research has established a strong connection between neurotransmitters like serotonin and dopamine and depression. Furthermore, inflammatory cytokines have been shown to stimulate astrocytes to release glutamate and reduce the expression of glutamate transporters in astrocytes, potentially leading to excitotoxicity from glutamate [42,43]. Elevated CNS glutamate levels are a key feature of depression.

Physical activity reduces inflammation through various mechanisms, aiding the recovery and prognosis of breast cancer patients. One of the main ways physical activity reduces inflammation is by modulating cytokines. A study of breast cancer patients showed that regular exercise significantly reduced local inflammation in breast tissue, especially the expression of TNF-α. Exercise also promoted the expression of the anti-inflammatory factor IL-10 [44]. IL-6 is typically categorized as a pro-inflammatory cytokine because it is secreted by T cells and macrophages, activating the immune system and inducing inflammation [45]. IL-6 levels increase exponentially after exercise (up to 100-fold) but rapidly decline after exercise [3]. Studies show that contracting muscles release IL-6 into the bloodstream. TNF-α and IL-1β are considered classic pro-inflammatory cytokines, released during cell damage. Through their actions, they activate immune cells and stimulate a pro-inflammatory response by increasing systemic prostaglandins [46]. However, IL-6 from skeletal muscles and IL-6-induced acute-phase proteins have anti-inflammatory and immune-suppressive effects, promoting anti-inflammatory responses by stimulating the production of IL-10- and IL-1-receptor antagonists and inhibiting TNF-α [47]. Experiments have demonstrated that IL-6 inhibits TNF-α production in cultured human monocytes and monocytic cell lines, and this relationship was confirmed in IL-6 knockout mice and wild-type mice treated with anti-IL-6, where TNF-α levels in circulation were significantly elevated [48]. IL-1RA is primarily secreted by monocytes and macrophages and inhibits the pro-inflammatory effects of IL-1β [49]. IL-10, primarily produced by regulatory T cells, downregulates adaptive immune responses, reducing tissue damage from inflammation. Specifically, IL-10 can reduce the expression of MHC molecules, intercellular adhesion molecule 1 (ICAM1), and co-stimulatory molecules CD80 and CD86 on antigen-presenting cells, promoting the differentiation of DCs into types with low MHC II, CD80, and CD86 expression. Moreover, IL-10 can downregulate or completely inhibit the expression of various pro-inflammatory cytokines and soluble mediators, weakening the ability of effector T cells to sustain inflammation [50]. Thus, IL-10 is a potent promoter of anti-inflammatory states [51]. Gleeson et al. pointed out in their review that chronic exercise training can reduce fat tissue inflammation, potentially by inhibiting macrophage infiltration and accelerating the phenotypic transition of macrophages from pro-inflammatory M1 macrophages to anti-inflammatory M2 macrophages [52], thus alleviating chronic inflammation in exercisers.

Acute and chronic physical activity have wide-ranging physiological effects on immune cells. Moderate physical activity is associated with increased T cell proliferation [53,54], reduced levels of senescent T cells [55], lower levels of inflammatory cytokines [56], and greater NK cell cytotoxicity [57,58]. Exercise also reduces inflammation by modulating circulating adipokines. The accumulation of fat tissue increases the circulation of adipokines, such as elevated IL-6, TNF-α, and leptin [59]. Exercise can counteract this inflammation by reducing fat tissue accumulation [60]. Some evidence suggests that exercise may affect monocyte morphology, reducing the expression of toll-like receptor (TLR) subtypes (e.g., TLR4) that regulate inflammatory responses [61]. Through these pathways, exercise can stimulate physiological changes that create a lasting anti-inflammatory environment.

Clearly, physical activity improves depressive symptoms through its effects on inflammation. A large cross-sectional analysis based on NHANES data showed a positive correlation between depression and inflammatory markers, whereas physical activity (PA) was negatively correlated with both inflammatory markers and depression. The greatest reduction in depression risk occurred at PA levels between 1200 and 1722 MET-min/week. Inflammatory markers mediated the potential impact of physical inactivity on depression, ranging from 1.72% to 6.25% [62]. A study on university students’ campus exercise also confirmed this point, suggesting that moderate physical activity may improve depression by reducing TNF-α [63].

### 2.2. Neuroplasticity

Neuroplasticity refers to the brain’s ability to adapt and change its structure and function in response to external stimuli, primarily through alterations in neural connectivity, the formation of new synapses, and the reorganization of neurons. It is a crucial mechanism for learning, memory, and adapting to environmental changes. Significant changes in neuroplasticity have been observed in certain brain regions of patients with depression. The hippocampus, a structure with a high density of glucocorticoid receptors, has been found to shrink by 10–20% in patients with severe depression according to imaging studies [64,65,66]. However, this reduction is partially reversed after antidepressant treatment [67]. A decrease in the size of neurons in key hippocampal subregions and a reduction in neural fiber networks may be one of the reasons for the hippocampal volume reduction in depressed patients [68]. The prefrontal cortex (PFC)—which is primarily involved in cognition, working memory, and inhibitory control over brain regions related to fear and emotional responses—also shows a significant volume reduction in patients with severe depression, as evidenced by brain imaging studies [67,69,70]. Furthermore, reductions in neuron soma size have been observed, suggesting a decline in dendritic branching and complexity [71,72]. There is also a decrease in the number of astrocytes and oligodendrocytes [73,74], as glial cells provide metabolic support for neurons and regulate neurotransmitter activity. Therefore, the atrophy, damage, and dysfunction of PFC neurons may be related to a reduction in glial cell populations. Objective brain stimulation tests have demonstrated that, compared to healthy controls, individuals with depression exhibit reduced neuroplasticity in the motor cortex [75]. Breast cancer patients, who experience immense psychological stress during diagnosis, treatment, and recovery, also exhibit impaired neuroplasticity, particularly in brain regions associated with emotion regulation. Stress-induced neuroplasticity deficits are considered a significant driver of depressive symptoms in breast cancer patients. Research has shown that stress-related neuroplasticity damage can be partially restored through antidepressant treatment and neuromodulatory interventions [76]. For example, transcranial direct current stimulation (tDCS) has been shown to improve motor cortex neuroplasticity and is associated with emotional improvement; however, the improvement in neuroplasticity does not always directly correlate with mood enhancement, possibly due to differential responses in various brain regions [77].

Brain-Derived Neurotrophic Factor (BDNF) is a key protein that promotes neuron survival, development, and functional maintenance and is closely linked to neuroplasticity. BDNF is highly expressed in brain regions such as the hippocampus and PFC, which are involved in emotional and cognitive functions. By promoting neuronal growth, synapse formation, and synaptic plasticity, BDNF helps maintain the health of neural networks. Numerous studies have indicated that reduced BDNF levels are closely associated with the onset of depression. A decline in BDNF may be an important biological marker of depressive symptoms. After antidepressant treatment, BDNF levels often increase, reflecting a partial recovery of neuroplasticity [78]. Additionally, the Val66Met polymorphism of the BDNF gene has been found to be associated with inflammation-related depressive symptoms. Individuals carrying the Met allele tend to have lower levels of neuroplasticity and are more prone to cognitive depressive symptoms in inflammatory contexts [79].

Chemotherapy-induced cognitive impairment is a common side effect in breast cancer patients and may be linked to impaired neuroplasticity. A study showed that exercise could prevent cognitive decline by improving hippocampal neuroplasticity. A low-intensity running exercise was found to alleviate chemotherapy-induced hippocampal dysfunction and restore neurogenesis [80]. Similarly, patients undergoing chemotherapy often experience significant reductions in hippocampal volume, which is thought to contribute to cognitive dysfunction. Exercise has been shown to have neuroprotective effects by promoting neurogenesis and enhancing hippocampal plasticity, suggesting that physical activity could mitigate chemotherapy-induced cognitive deficits [81]. Animal studies have shown similar results. Mice that engaged in voluntary wheel running exhibited a significant increase in brain BDNF levels and enhanced synaptic plasticity through BDNF signaling pathways [82]. Acute aerobic exercise enhances neuroplasticity, particularly in the motor cortex. Exercise supports neuroplasticity by promoting synaptic connections between neurons and enhancing long-term potentiation (LTP) [83]. Even a brief bout of aerobic exercise can significantly enhance neuroplasticity in the motor cortex. This enhanced plasticity aids in motor learning and brain function recovery [84]. A review summarizing the behavioral and neural outcomes of exercise interventions across different age groups in humans showed that exercise may trigger processes that promote neuroplasticity, thereby enhancing an individual’s ability to adapt to new demands through behavioral responses [85]. This concept has been demonstrated in animal studies. A study using diffusion tensor imaging (DTI) to monitor hippocampal microstructural changes in mice after voluntary wheel running found that exercise significantly increased hippocampal volume and fractional anisotropy, suggesting adaptive changes in hippocampal microstructure and supporting the concept of exercise-induced neuroplasticity [86]. Different exercise regimens have varying effects on brain neuroplasticity. One study compared the effects of two different treadmill exercise regimens on synaptic proteins and glutamate receptors in the rat motor cortex, striatum, and cerebellum. The study found that different exercise frequencies affected synaptic remodeling in different ways. Intermittent exercise increased the expression of presynaptic proteins, while continuous exercise enhanced the expression of postsynaptic AMPA receptors [87].

Numerous studies in both humans and animals indicate that the onset of depressive symptoms is linked to a deficit in neuroplasticity, and physical activity can enhance neuroplasticity in the brain. A three-week exercise program significantly improved LTP levels in the brain, indicating that exercise can restore neuroplasticity. More importantly, the restoration of neuroplasticity is closely associated with the alleviation of depressive symptoms, suggesting that enhanced neuroplasticity directly contributes to mood improvement [88]. Yoga and meditation not only reduce the severity of depression but also enhance the brain’s recovery capacity by increasing neuroplasticity markers such as BDNF [89]. Furthermore, exercise appears to activate mitochondrial pathways and promote neuroplasticity. An animal study found that exercise stimulated mitochondrial activity in the brain and increased resistance to rotenone, a complex I inhibitor. Post-exercise, there was increased mRNA expression of BDNF, Glial Cell-Derived Neurotrophic Factor (GDNF), TFAM (mitochondrial transcription factor A), and Ndufa6 (mitochondrial complex I subunit), as well as increased phosphorylation of the cAMP response element-binding protein, suggesting that exercise reduces anxiety-like behaviors and may exert antidepressant-like effects in mice, promoting neuroplasticity and improving mood [90]. Physical activity increases gray matter volume in the hippocampus and PFC, both of which are crucial for emotion regulation. In depression, these regions often exhibit atrophy but exercise can promote brain function recovery by enhancing their volume [91]. Additionally, exercise improves brain connectivity by regulating neurotransmitter-release and synaptic-plasticity mechanisms, enhancing emotion regulation and cognitive abilities [92]. While there is ample evidence supporting the link between neuroplasticity, depression, and exercise, more definitive biological evidence is still needed.

### 2.3. Glutamate Metabolism

**Figure 2:**
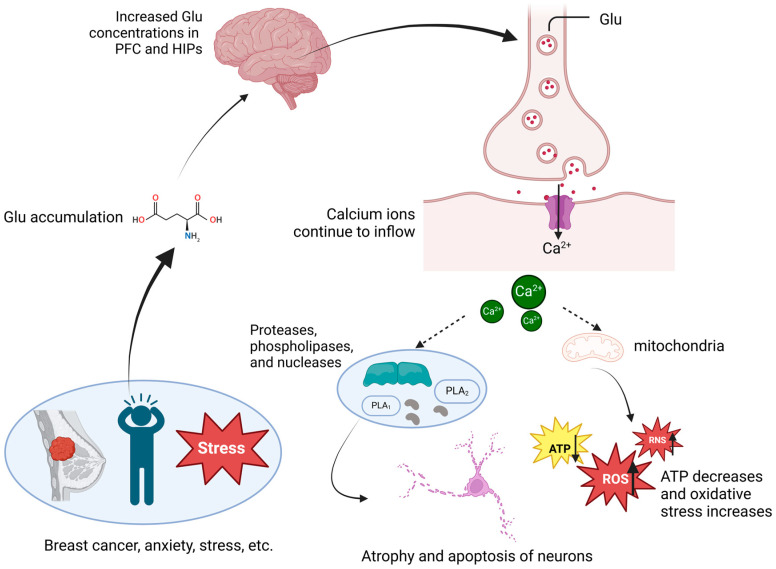
The biological mechanisms by which glutamate metabolism leads to depression. Abbreviations: ATP, adenosine triphosphate; ROS, reactive oxygen species; RNS, reactive nitrogen species; Glu, glutamate; PFC, prefrontal cortex; PLA1, phospholipase A1; PLA2, phospholipase A2. The figure was created in BioRender at the following site: https://BioRender.com.

Glutamate is the primary excitatory neurotransmitter in the CNS, playing a critical role in regulating synaptic transmission, neuronal plasticity, learning, and memory, among other essential functions. In individuals with depression, glutamate levels in the brain are often abnormally elevated, particularly in areas involved in emotion regulation, such as the PFC and hippocampus. Chronic exposure to stressful environments can lead to excessive glutamate release, which may result in dendritic atrophy and synaptic remodeling, ultimately affecting cognitive function, emotion regulation, and behavior, and contributing to or exacerbating depressive symptoms [93]. Both glutamate and its precursor, glutamine, are significantly elevated in the cerebrospinal fluid of individuals with depression, and their levels are positively correlated with the severity of depressive symptoms [94]. Glutamate exerts its effects in the CNS primarily through three receptor types: N-methyl-D-aspartate (NMDA) receptors, α-amino-3-hydroxy-5-methyl-4-isoxazolepropionic acid (AMPA) receptors, and metabotropic glutamate receptors (mGluRs). Glutamate mediates the calcium ion influx into neurons via NMDA receptors, triggering downstream signaling pathways that enhance postsynaptic density and synapse formation, thereby promoting neuroplasticity [95]. In individuals with depression, excessive glutamate continuously activates NMDA receptors, leading to a prolonged calcium influx into neurons. This influx activates destructive enzymes, including proteases, phospholipases, and nucleases, resulting in neuronal damage [96]. The excessive calcium entering the mitochondria disrupts their normal function, interfering with energy metabolism and reducing ATP production. Mitochondrial dysfunction also triggers oxidative stress and the production of ROS, further exacerbating cellular damage [97]. Calcium overload also leads to the excessive production of ROS and RNS, inducing programmed cell death and contributing to neuronal toxicity [96]; the specific process is shown in Figure 2. Animal model studies have shown that under chronic stress conditions, increased glutamate release leads to structural damage in neurons, particularly dendritic atrophy and synapse loss. Glutamine, a non-neuroactive molecule synthesized by astrocytes, plays a crucial role in the glutamate–glutamine cycle. After glutamate is released into the synaptic cleft, it is taken up by astrocytes and converted into glutamine. Glutamine is then transported back into the synapse where it is reabsorbed by neurons and converted into glutamate for neurotransmission. In individuals with depression, a reduction in glutamine synthetase (GS) activity leads to an abnormal increase in glutamate concentrations in the synaptic cleft. This abnormal accumulation may induce neurotoxicity, further impairing neuronal function and exacerbating depressive symptoms [98,99]. Ketamine, an NMDA-receptor antagonist, has demonstrated rapid and significant antidepressant effects, particularly in the treatment of treatment-resistant depression [100]. After a single intravenous injection of ketamine, depressive symptoms in patients significantly improve within hours [101]. Additionally, novel drugs such as AMPA-receptor modulators, mGluR antagonists, and excitatory amino-acid transporter (EAAT) regulators have shown promising antidepressant effects in clinical and animal models. These drugs modulate glutamate release and clearance through various mechanisms, reducing glutamate-mediated neurotoxicity and alleviating depressive symptoms [102]. Glutamate not only regulates neuroplasticity through direct receptor activation but also influences neuronal development and survival through interactions with neurotrophic factors such as BDNF. Glutamate stimulates the release of BDNF, which, in turn, enhances synaptic transmission and plasticity by upregulating the expression of glutamate receptors. This feedback loop helps maintain neuroplasticity in the brain [103].

The interaction between inflammation and the glutamate system is also a critical mechanism in the development of depression. Inflammatory factors can modulate glutamate metabolism through astrocytes and microglia. In chronic inflammatory states, increased inflammatory factors lead to glutamate accumulation in the brain, impairing glutamate clearance mechanisms and intensifying neuronal damage. Magnetic resonance spectroscopy (MRS) studies have shown that inflammatory markers in individuals with depression correlate positively with glutamate levels in the basal ganglia and anterior cingulate cortex [104]. The effects of physical activity on glutamate metabolism are complex and closely linked to the glutamate–glutamine cycle. After intense physical activity, the levels of glutamate and glutamine in the brain increase significantly, particularly in the cerebellum and hippocampus [105]. In mouse models, prolonged voluntary exercise notably increases the levels of glutamate-related postsynaptic density proteins, such as PSD-95, SAP-97, and GRIP-1, in the cerebral cortex. Exercise also increases the phosphorylation of NMDA receptors and enhances the binding of MK-801—a specific ligand that binds to open NMDA channels—by 51%. This process regulates NMDA receptor activation and ion channel opening, increasing the receptor’s responsiveness to glutamate and improving synaptic signaling [106]. Aerobic exercise, such as running, promotes hippocampal neurogenesis, where glutamate, in conjunction with other neurotransmitters like acetylcholine, stimulates the development of new neurons, ultimately improving memory and learning abilities [107]. These increases in protein levels and changes in synaptic activity may be linked to the cognitive and emotional improvements associated with exercise.

Although the elevation of glutamate levels following exercise may be beneficial, the activation of glutamate receptors, such as NMDA and AMPA, can lead to excessive excitability and induce fatigue [108]. In extreme cases, the over-release of glutamate may cause neuronal damage, and it is crucial to maintain balanced glutamate activity during exercise [109]. During the recovery phase following exercise, glutamate levels in the synaptic cleft significantly increase. Astrocytes play a vital role in preventing the neurotoxicity associated with glutamate accumulation by accelerating its reuptake via increased glutamine synthesis, thereby alleviating exercise-induced excitotoxicity [110]. Elevated glutamate levels can cause neuronal atrophy and contribute to depression, while lower levels may activate adaptive stress responses that protect neurons from severe stress [111]. Moderate exercise enhances glutamate homeostasis, counteracting the glutamate imbalance caused by chronic stress. This regulatory effect helps restore brain function and alleviate the cognitive and emotional symptoms associated with depression.

While there is currently limited direct evidence for exercise improving depression via glutamate pathways, clinical and animal studies involving physical activity have demonstrated improvements in depressive symptoms and the regulation of glutamate metabolism [106,107]. Exercise may also enhance neuroplasticity by increasing postsynaptic density proteins and the phosphorylation of NMDA receptors, ultimately improving depression [108]. The interaction between glutamate and inflammation also warrants further investigation. Inflammation increases glutamate release and accumulation, exacerbating depressive symptoms. Regular exercise can effectively reduce chronic low-grade inflammation [112], modulating glutamate transmission and metabolism, which may improve depressive symptoms [104,113]. The interplay between glutamate and BDNF may optimize glutamatergic signaling, reduce oxidative stress, and protect neurons, thereby improving mood disorders like depression [103]. Glutamate metabolism may be one of the pathways through which exercise improves depression, however, more research is needed to validate this hypothesis.

### 2.4. Other Factors

#### 2.4.1. Oxidative Stress

Oxidative stress refers to the excessive generation of ROS or RNS in the body that exceeds the capacity of the antioxidant defense system to neutralize them, leading to cellular damage. Free radicals are highly reactive molecules generated during metabolic processes, and are capable of interacting with proteins, lipids, DNA, and other molecules, resulting in cellular dysfunction and tissue injury. Depressed individuals often exhibit elevated levels of oxidative stress and a decline in antioxidant capacity. The changes in oxidative stress markers in depressed patients are complex and multifaceted. Most studies indicate a significant increase in oxidative stress markers and a notable decrease in antioxidant capacity in these patients [114]. At the same time, in depressed patients, key antioxidants such as serum uric acid, zinc, and vitamin C as well as redox enzyme levels are reduced. Additionally, oxidative damage markers and oxidative DNA damage markers, such as malondialdehyde (MDA), F2-isoprostanes, and 8-OHdG, are significantly elevated [115,116]. Antidepressant treatment has been shown to partially improve oxidative stress levels. Studies have found that after treatment with antidepressants such as sertraline or bupropion, oxidative stress markers (e.g., MDA) decreased, while levels of antioxidants (e.g., serum zinc and uric acid) increased [117]. However, some studies also suggest that antidepressant therapy may have little to no significant impact on certain oxidative stress markers, or may even lead to an increase in the levels of some markers [118].

Increased oxidative stress is also associated with the development and progression of breast cancer. Research has shown that oxidative stress markers, such as SOD and GPX, are significantly elevated in breast cancer patients, while catalase (CAT) activity is notably reduced [119]. Estrogen contributes to increased oxidative stress in breast cancer cells by inhibiting uncoupling proteins [120]. Clinical studies have also shown that in breast cancer patients with depression, depressive symptoms are significantly associated with a decrease in antioxidant levels [121].

Physical activity can regulate the oxidative stress state in cancer patients, particularly by enhancing the activity of antioxidant enzymes and reducing oxidative damage markers. Moreover, it decreases oxidative damage markers in various cancer populations [122]. However, the effects of exercise vary across different groups. Outdoor activities, such as long-distance hiking, can improve the antioxidant capacity of breast cancer patients but the response differs between men and women. Women show a significant enhancement in antioxidant capacity, while men exhibit an exacerbation of oxidative stress [123]. A study on vascular depression found that oxidative-stress-induced damage to endothelial cells exacerbated depressive symptoms, while exercise alleviated these symptoms by reducing oxidative damage [124]. Exercise can also regulate key metabolic pathways by activating specific signaling pathways, such as the PGC-1α pathway, to reduce harmful byproducts of oxidative stress. This mechanism reduces the likelihood of oxidative-stress-induced damage to the brain and improves the ability of depressed individuals to cope with stress [125]. In animal experiments, oxidative stress in the hippocampus and amygdala of rats significantly increased both before and after depression modeling, manifesting lipid peroxidation, protein oxidation, and decreased antioxidant enzyme activity; however, pre-emptive aerobic exercise significantly reduced these oxidative stress markers and improved the rats’ memory and behavior [126]. Another animal study found that chronic stress significantly reduced mitochondrial function, BDNF levels, and SOD activity in the brain of rats while increasing oxidative stress markers; exercise pre-treatment effectively restored mitochondrial function, significantly increased SOD activity and BDNF levels, and alleviated oxidative stress [127]. Comparisons with drug treatments revealed that physical activity, as a therapeutic approach, demonstrated similar or even superior effects in reducing oxidative stress and improving cardiac function, with reduced oxidative stress levels and enhanced mitochondrial function in heart tissue. These animal studies suggest that physical activity can effectively alleviate depressive-like behaviors by enhancing the antioxidant system, restoring mitochondrial function, and upregulating neurotrophic factors such as BDNF. Therefore, physical activity, as a non-pharmacological intervention, offers significant antidepressant effects. However, clinical validation—particularly in cancer populations—remains limited, and further studies are needed to understand how exercise influences oxidative stress in patients with depression.

#### 2.4.2. Neuroendocrine System

The relationship between depression and the neuroendocrine system is a complex domain involving multiple biochemical pathways. Dysregulation of the neuroendocrine system may play a crucial role in the pathogenesis of depression. The HPA axis is a key system that regulates the body’s stress response. This axis involves both forward stimulation and feedback inhibition loops between the brain, pituitary, and adrenal glands, controlling the production of glucocorticoids. Cortisol, secreted by the adrenal glands, primarily exerts its effects through mineralocorticoid receptors in the hippocampus [128]. Feedback from the pituitary and brain regions is mediated by glucocorticoid receptors [129,130]. Early life stress often leads to neuroendocrine dysfunction, resulting in HPA-axis dysregulation. This can cause abnormal levels of cortisol and contribute to various physiological and psychological symptoms associated with depression [131]. Multiple studies indicate that the HPA axis in depressed individuals is often in a state of hyperactivation. Meta-analyses have shown that higher cortisol levels are associated with persistent depressive symptoms, and the relationship with somatic symptoms is stronger than that with cognitive and emotional symptoms [132]. For example, breast cancer patients undergoing chemotherapy experience elevated cortisol levels after treatment, which exacerbate depressive symptoms [133]. In contrast, psychotherapy significantly reduces cortisol levels, improving both depressive and anxiety symptoms while enhancing immune function [134]. Patients with depression often exhibit impaired negative feedback on cortisol. Dexamethasone is commonly used to assess HPA-axis function in depression. Some depressed patients fail to show the expected cortisol suppression after dexamethasone administration. Severe depression is often characterized by heightened sensitivity of the HPA axis and behavioral hypersensitivity, with elevated baseline cortisol levels. This is believed to be partly due to desensitization of the negative feedback mechanisms of the HPA axis, leading to persistently elevated cortisol levels. This phenomenon is more prevalent in patients with recurrent depression and is associated with a higher risk of relapse [135]. In animal studies, male rats exposed to chronic unpredictable stress exhibit increased excitatory input and decreased inhibitory input on neurons in the paraventricular nucleus (PVN), suggesting heightened sensitivity of the HPA axis and a failure of normal negative feedback mechanisms [136]. Cortisol not only mediates the stress response but also enhances hippocampal function. However, under prolonged and inflammatory stress conditions, it can damage hippocampal neurons, leading to a reduction in hippocampal volume and dendritic branching in depressed patients. Antidepressants can effectively upregulate glucocorticoid receptor (GR) expression and restore normal GR function, allowing the HPA axis to appropriately respond to negative feedback [137]. Furthermore, depressed patients often exhibit disrupted circadian rhythms of cortisol secretion. Normally, cortisol follows a distinct diurnal rhythm, peaking in the morning and dropping to its lowest level at night; however, this rhythm is frequently disrupted in individuals with depression, and such abnormalities are linked to the severity, duration, and treatment response of the disorder [138]. Moreover, cortisol dysregulation may lead to sleep disturbances, further exacerbating depressive symptoms [139].

Physical activity has profound effects on the neuroendocrine system, triggering a range of complex neuroendocrine responses that help maintain homeostasis by regulating the body’s stress system, immune function, and energy metabolism. As stated by Leal-Cerro et al., physical activity—especially high-intensity exercise—significantly activates the HPA axis, stimulating the secretion of adrenocorticotropic hormone (ACTH), which in turn stimulates the adrenal cortex to release cortisol for regulating glucose metabolism, blood sugar levels, and immune responses. Additionally, exercise also triggers the release of adrenaline and noradrenaline from the adrenal medulla, accelerating heart rate, increasing blood pressure, and enhancing energy supply [140]. Chronic stress is often accompanied by prolonged activation of the HPA axis and excessive secretion of glucocorticoids. This persistent hormone release may lead to metabolic disorders, such as insulin resistance, central obesity, and depression. Regular exercise helps reduce the excessive response of the HPA axis and mitigates the adverse effects of chronic stress on metabolism and mental health [141]. Physical activity triggers a series of neuroendocrine adaptations that enhance the body’s ability to cope with future stressors. Through repeated exercise-induced stress, the neuroendocrine system gradually learns to attenuate its response to the same stimuli, thus reducing excessive activation of the HPA axis [142].

In the breast cancer patient population, strong evidence has been established for the beneficial effects of physical activity on improving depressive symptoms. Mind–body integrative exercises, such as yoga, have been shown to significantly reduce stress, regulate cortisol secretion, and increase the number of natural killer (NK) cells. Patients experience improvements in sleep quality, mood, and immune function [143]. Additionally, a combination of aerobic exercise and low-intensity exercise has been shown to significantly improve HPA-axis function in breast cancer patients, reduce depressive symptoms, and have a positive impact on long-term survival [144]. Exercise has been found to reduce the 24-hour urinary excretion of cortisol and adrenaline in adolescent females with depression [145]. Animal studies have also demonstrated that sustained physical activity can effectively reverse the cortisol increase and the reduction in hippocampal neurotransmitter expression caused by stress, thereby alleviating depressive symptoms [146]. Overall, physical activity has a powerful regulatory effect on the HPA axis, with significant benefits observed in depressive populations.

#### 2.4.3. Monoamine Neurotransmitters

**Figure 3:**
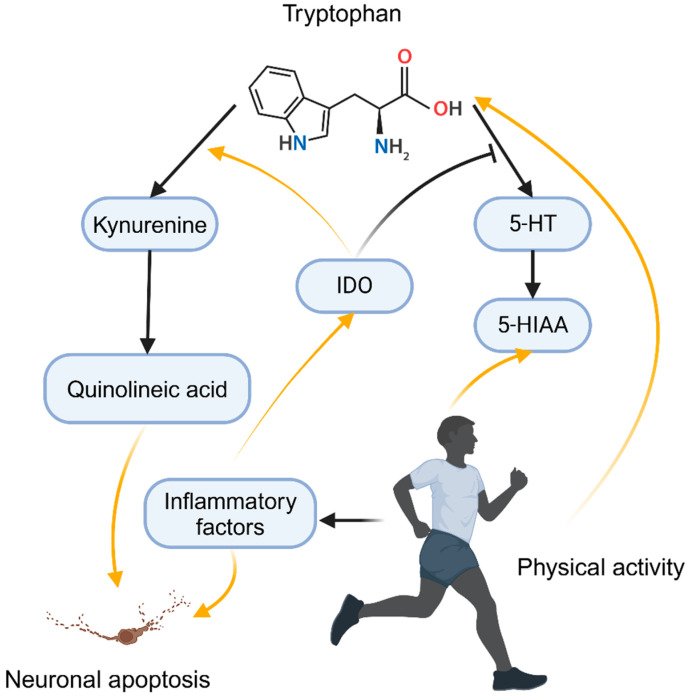
Exercise ameliorates depression by influencing the neuroendocrine system. Abbreviations: 5-HT, 5-hydroxytryptamine (serotonin); 5-HIAA, 5-hydroxyindoleacetic acid; IDO, indoleamine 2,3-dioxygenase. The figure was created in BioRender at the following site: https://BioRender.com.

Monoamine neurotransmitters, such as serotonin (5-hydroxytryptamine, 5-HT), noradrenaline, and dopamine, play a central role in mood regulation. Serotonin (5-HT), in particular, is of significant interest as dysregulation of serotonin function is associated with various psychiatric disorders, especially depression and anxiety [147]. Tryptophan, a precursor of serotonin, plays a crucial role in serotonin synthesis, with its intake and transport in the brain directly influencing serotonin levels. Serotonin synthesis begins with the essential amino acid tryptophan. A deficiency in tryptophan or impairments in its transport across the blood–brain barrier can lead to reduced serotonin synthesis, triggering depressive symptoms [148]. In conditions of immune activation and inflammation, pro-inflammatory cytokines such as IFN-γ and TNF-α activate indoleamine (IDO), which diverts tryptophan metabolism from serotonin to kynurenine. The activation of this pathway not only reduces the production of tryptophan but also leads to the accumulation of kynurenine metabolites such as quinolinic acid, which has neurotoxic effects. Quinolinic acid can exacerbate neuronal damage through excitotoxicity, thereby triggering or worsening depressive symptoms [149,150]. Garrison et al. studied the effects of quinolinic acid (QUIN) on microglial cells, finding that QUIN activates NMDA receptors, triggering oxidative stress and neuronal death. Inhibition of QUIN production attenuated the inflammatory response [151], as illustrated in Figure 3. In cancer populations, IDO overexpression is common, and in breast cancer, IDO expression often co-occurs with programmed death ligand 1 (PD-L1) expression, correlating with poor prognosis [152,153].

Physical activity, particularly endurance exercise, can significantly enhance tryptophan uptake, leading to increased serotonin synthesis. Animal studies have shown that exhaustive training raises serotonin levels in the hypothalamus [154]. During exercise, the release of free fatty acids increases, which releases tryptophan bound to albumin, thereby increasing the availability of “free tryptophan” for brain transport. This elevated tryptophan uptake leads to increased serotonin production, facilitating central nervous system regulation [155]. Following exercise, levels of 5-hydroxyindoleacetic acid (5-HIAA)—a serotonin metabolite—increase, indicating enhanced serotonin metabolism. This suggests that exercise not only boosts serotonin synthesis but also promotes its metabolism and clearance, preventing the negative effects of excessive serotonin levels [156].

Physical activity significantly promotes neurogenesis, particularly in the hippocampus, by enhancing serotonin signaling. Neurogenesis is closely linked to antidepressant effects, and regular aerobic exercise increases the generation of new neurons, improving brain plasticity and cognitive function. This process is primarily mediated by serotonin-regulated signaling pathways, where exercise activates 5-HT1A receptors, promoting neuronal differentiation and survival [157,158]. Long-term aerobic exercise training enhances serotonin receptor function in the brain, improving mood and stress resilience. This adaptation not only improves the efficiency of serotonin signaling but also enhances both mood and physical performance in athletes. A study investigated the effects of aerobic walking exercise on elderly breast cancer patients undergoing hormone therapy. The results showed that the exercise group had a significant increase in serotonin levels, and depressive symptoms improved [159]. In an animal study, depressed rats exhibited behavioral improvements following exercise. Compared to the control group, the exercise group showed a significant increase in serotonin, dopamine, and noradrenaline levels in the hippocampus [160]. In another study, chronic unpredictable mild stress (CUMS) induced depressive-like behavior in mice, accompanied by an increase in inflammation and IDO (indoleamine 2,3-dioxygenase) activity. However, aerobic exercises such as swimming significantly alleviated the stress-induced depressive symptoms, reduced pro-inflammatory cytokines and IDO activity, and increased serotonin levels in the brain [161].

## 3. Discussion

Depression is a complex mental disorder characterized by a wide range of symptoms and high heterogeneity, which makes diagnosis and treatment particularly challenging. Despite the broad applicability of the diagnostic criteria for major depressive disorder (MDD) defined by the Diagnostic and Statistical Manual of Mental Disorders (DSM), there are significant individual differences in symptom manifestation and treatment responses [162]. In many cases, traditional pharmacological and psychotherapeutic treatments have proven insufficient. This is largely due to the complex interplay of biological mechanisms underlying depression.

In the complex mechanisms of depression, the role of inflammation seems particularly significant. First, inflammatory factors can activate IDO, thereby affecting the production of serotonin [40]. Inflammatory cytokines impair tetrahydrobiopterin, affecting the metabolism of monoamine neurotransmitters [41]. In the glutamate system, inflammatory cytokines can stimulate astrocytes to release glutamate and reduce the expression of glutamate transporters in astrocytes, which leads to increased excitotoxicity of glutamate [42,43]. Inflammatory factors also reduce the levels of neurotrophic factors like BDNF, further affecting neuroplasticity. Additionally, inflammation affects the HPA axis, with chronic inflammation activating the HPA axis and leading to abnormal cortisol production, which in turn causes mood disorders. The relationship between inflammation and oxidative stress is also complex; excessive ROS production activates pathways like NF-κB and MAPK, promoting the production of pro-inflammatory molecules. This can also cause cellular damage and the generation of pro-inflammatory molecules like MDA, which further exacerbate the inflammatory response [6,163]. It can be said that the overactivation of ROS and the inflammatory system are key contributors to the pathogenesis of depression.

As previously detailed, the production of inflammation and its contribution to depression have been well-explained. However, we must also acknowledge the heterogeneity of inflammation as a significant manifestation in depressed patients. It is undeniable that the average levels of various inflammatory mediators are often higher in depressed populations compared to non-depressed groups but the increase in pro-inflammatory cytokines and other inflammatory factors observed in depression is much more moderate than what is seen in autoimmune or infectious diseases. Research on depressed patients has found that they often possess traits that lead to elevated inflammation, such as body mass index and gender. After controlling for these factors, some studies suggest there are no significant differences in inflammation traits between depressed and non-depressed groups. In response to this phenomenon, Raison and colleagues pointed out in their review that the elevated levels of multiple inflammatory biomarkers in depressed populations are due to a higher number of individuals exhibiting these increases compared to the control group. Oxidative stress also repeatedly appears in the complex mechanisms of depression. Increased ROS production across various pathways induces overactivation of the HPA axis, leading to elevated glucocorticoids, which in turn increase mitochondrial activity. The superoxide hydroxyl radicals generated in this process can cause further oxidative damage [164,165]. Recent studies have been exploring the relationship between oxidative stress and serotonin in the context of depression. Tryptophan can be metabolized into kynurenine through inflammatory cytokines or reactive oxygen species, further generating other neurotoxic substances that damage neuronal function [6,166]. Additionally, drugs targeting both serotonin and oxidative stress, such as selenium-modified fluoxetine derivatives, have shown significant positive effects on depressive symptoms [167]. Mirtazapine and L-tryptophan also counteract peroxide-induced cellular stress [168]. In summary, oxidative stress reduces serotonin production and generates toxic substances. Serotonin metabolites can partially alleviate oxidative stress, and drugs targeting both processes show significant positive effects. Changes in neuroplasticity are also closely related to other biological mechanisms of depression. Glutamate can stimulate the release of neurotrophic factors like BDNF, and BDNF and other neurotrophic factors upregulate the expression of glutamate receptors, enhancing synaptic transmission and plasticity [103]. Serotonin signaling also affects BDNF expression and promotes neurogenesis [169]. Chronic stress leads to dysregulation of the HPA axis and excessive secretion of glucocorticoids, which disrupt the balance between pro-inflammatory cytokines and neurotrophic factors [170,171]. Elevated glucocorticoid levels inhibit neurogenesis and promote neuronal atrophy.

First, the diversity of depressive symptoms is intricately linked to the interactions between biological processes. For example, chronic inflammation can lead to the excessive activation of glutamate, which in turn disrupts neuronal function and neuroplasticity [172]. Chronic inflammation is typically aggravated by stress, which triggers microglial cells in the brain to release pro-inflammatory cytokines. This cascade of events contributes to cognitive impairments and emotional disturbances that are commonly observed in depression [173]. Additionally, a deficiency in antioxidants can exacerbate oxidative stress, further impairing the function of monoamine neurotransmitters. Chronic stress also leads to the activation of the HPA axis, increasing cortisol levels and promoting inflammatory responses [170]. These interconnected mechanisms may help explain the variability in symptom presentation and the differential responses to treatments seen in patients. The situation becomes even more complex in patients with breast cancer. During the diagnosis and treatment process, breast cancer patients endure considerable psychological stress, inflammatory responses, and challenges to their immune systems, all of which intensify the severity of their condition. Research indicates that approximately 32% of breast cancer patients experience depression, with the incidence rising to 50% in early-stage cases [174,175]. Furthermore, hormonal fluctuations are also recognized as key triggers for depression [176]. Even more concerning is the possibility that depressive symptoms, through the enhancement of inflammation and immune responses, may accelerate tumor progression [174,177]. Thus, it is crucial to explore the manifestation and impact of depression in breast cancer patients.

Increasing physical activity has been shown to be an effective intervention for alleviating depressive symptoms in breast cancer patients. Moderate exercise not only helps reduce chronic inflammation and improve oxidative stress but also enhances neuroplasticity, benefiting the neuroendocrine system and neurotransmitter metabolism. It is particularly important to tailor the type of physical activity to the individual’s needs, with options such as walking, yoga, and Tai Chi being highly suitable for breast cancer patients. To further understand how physical activity affects depressive symptoms in this group, large-scale, multi-center clinical studies are urgently needed. These studies should investigate the relationship between physical activity and depression, and explore changes in biological markers. Longitudinal studies that evaluate both the short-term and long-term efficacy of combined therapies will help develop more precise and personalized treatment strategies.

Although this review provides valuable insights into the impact of physical activity on depression in breast cancer patients, several limitations should be acknowledged. First, most of the studies included in this review are cross-sectional in nature, preventing the establishment of causal relationships between physical activity and depression. Second, the use of self-reported and clinician-reported measures in the studies may introduce response biases, particularly in self-report data, which could be influenced by participants’ emotional fluctuations and social desirability effects, potentially affecting the accuracy of the results. Moreover, some studies exhibited heterogeneity in terms of data collection, methodology, and sample selection, which may have contributed to inconsistencies in findings and reduced the strength of the evidence, ultimately impacting the reliability of the conclusions. Finally, while many studies have investigated the potential benefits of physical activity on depression, variations in sample characteristics and intervention protocols may lead to divergent results. Therefore, future research should employ longitudinal designs with more diverse and representative samples to better elucidate the long-term effects of physical activity on depression in breast cancer patients. Additionally, the use of multifaceted assessment methods and standardized intervention protocols will enhance the reliability and applicability of the findings.

## 4. Conclusions

Physical activity has a significant antidepressant effect in breast cancer patients, mediated through interactions between inflammation, oxidative stress, neuroplasticity, neuroendocrine regulation, monoamine neurotransmitters, and glutamate metabolism. These biological processes work synergistically to form an integrated antidepressant system in breast cancer patients. Increasing physical activity is an accessible and feasible intervention, with moderate-intensity activity already showing substantial benefits in alleviating depressive symptoms. Therefore, enhancing physical activity levels should be considered an essential strategy in the comprehensive management of breast cancer patients. A deeper understanding of these mechanisms can lead to more effective solutions for addressing depression and improving patients’ quality of life.

## 5. Future Directions

In conclusion, future research should continue to deepen our understanding of the biological mechanisms of depression, particularly in special populations such as breast cancer patients, and strive to develop personalized intervention strategies. By doing so, we will be better equipped to offer targeted and effective treatment options for individuals suffering from depression.
